# Predicting Perioperative Respiratory Adverse Events in Children Undergoing Elective Surgeries Under General Anesthesia Using COLDS Score: A Prospective Observational Study

**DOI:** 10.7759/cureus.84859

**Published:** 2025-05-26

**Authors:** Essam Mohamed H Hegazy, Houda Almusalhi, Suhrud Panchawagh, Abhijit Nair

**Affiliations:** 1 Anesthesia and Critical Care, Ibra Hospital, Ibra, OMN; 2 Anesthesiology, Ibra Hospital, Ibra, OMN; 3 Neurology, Smt. Kashibai Navale Medical College and General Hospital, Pune, IND

**Keywords:** adverse event, `anesthesia, ent procedures, pediatric, postoperative period, respiratory infection

## Abstract

Background and aims

Pediatric patients undergoing surgeries under general anesthesia (GA) who have a recent upper respiratory tract infection (URTI) pose a unique challenge for the anesthesiologists. We aimed to utilize the COLDS score as a pre-anesthetic risk assessment tool to predict the likelihood of perioperative respiratory adverse events (PRAEs) in children with URTI.

Methods and materials

After ethical approval, we prospectively collected data over six months from children undergoing various ear, nose, and throat (ENT) surgeries under GA. Children above one year undergoing elective ENT surgeries were included. Children less than one year, non-ENT surgical pediatric patients, and those undergoing emergency surgeries were excluded.

Results

A total of 270 patients were included in the analysis, among whom 25 (9.3%) experienced postoperative events. The COLDS score was able to effectively distinguish between patients who did and did not experience postoperative complications. The receiver operating characteristic (ROC) curve (AUC) of 0.92 (95% CI: 0.86-0.99) suggests a strong discriminatory ability of the score. The specificity of the model was high at 97.96% (95% CI: 95.9-99.6), suggesting a strong ability to correctly identify patients without events. The sensitivity was lower at 40.0% (95% CI: 20.7-60.0), i.e., only 40% of patients who experienced complications were detected by the score.

Conclusions

COLDS score can help in distinguishing patients who will and will not have PRAEs. But as the sensitivity is moderate, we recommend using the COLDS score along with a detailed clinical assessment based on the type of surgery planned.

## Introduction

Up to 30% of perioperative cardiac arrests in the pediatric population during anesthesia are caused by perioperative respiratory adverse events, which are a significant cause of morbidity and mortality [1,2]. Numerous studies have identified independent risk factors for perioperative respiratory adverse events, which includes a history of snoring, age less than 6 years old, a recent (<4 weeks) and ongoing upper respiratory infection (URI), asthma, prematurity, bronchopulmonary dysplasia, cystic fibrosis, pulmonary hypertension, infectious disease with significant impairment of the child's general condition (malaise, fever >38.5°C, bacterial superinfection), parental confirmation of the child's symptoms, the type of airway device used during surgery, the anesthesiologist's experience in pediatric anesthesia, and the type of surgery [3,4].

With an average of 6-8 URIs annually, upper respiratory tract infections are the most frequent preoperative comorbidity seen in children. A URI is present in about 30% of children who come in for elective surgery. Children with URIs are more likely to experience respiratory events, but the issues they face are usually minor and have no long-term effects. It is crucial to remember that there is a real chance of perioperative respiratory adverse events. Hyperreactivity in the bronchi may cause laryngospasm and bronchospasm, respectively, both of which can result in critically low oxygen levels [5-8].

In pediatric patients with perioperative respiratory adverse event risk factors, the choice of whether to proceed with anesthesia and surgery has generated controversy. While cancelling surgery altogether due to a URI reduces the risk of complications, it may not always be necessary and may have negative social, emotional, and financial effects on the child, family, and medical team. Risk stratification would be made possible by a thorough preanesthetic evaluation of the potential for perioperative respiratory adverse events.

The COLDS score is a pre-anesthetic risk assessment tool that was developed by Lee and August to predict the likelihood of perioperative respiratory adverse events (PRAEs) in children with upper respiratory tract infections (URIs) [9]. The score consists of five components: current symptoms (C), onset of URI (O), lung disease (L), device for airway management (D), and surgery type (S). Each component is assigned a value of 1, 2, or 5 based on the severity of the risk factor, and the total score ranges from 5 to 25. A higher score indicates a higher risk of PRAEs. This study aims to use the COLDS score as a prediction tool in children undergoing various surgeries under general anesthesia for perioperative respiratory adverse events.

## Materials and methods

This single-arm, observational, single-centre study was approved by the Centre of Studies and Research, Directorate General of Planning and Studies, North Sharqiya, Sultanate of Oman (proposal ID: MoH/CSR/23/27754). This study was conducted at the Department of Anesthesiology. Data gathered was reported per Strengthening the Reporting of Observational Studies in Epidemiology (STROBE) guidelines (www.strobe-statement.org). The objectives of this study were: 1) to assess the accuracy of the COLDS score in predicting PRAEs in children with URTIs, 2) investigating the relationship between the COLDS score and independent risk factors (e.g., symptoms, lung disease, type of surgery), and 3) analyzing demographic data for correlations with PRAEs.

The study population was children (above one year) undergoing various elective ENT surgeries under general anesthesia. The potential study participants were assessed either in the pre-anesthesia clinic or on the day of surgery, in the pre-anesthesia room, by history obtained from the parents, general/systemic examination, and by knowing the type of surgery/planned anesthesia management. Children more than one year scheduled for elective surgery (adenoidectomy, tonsillectomy, myringotomy, functional endoscopic sinus surgery (FESS), tongue-tie release, grommet insertion) were included. Children less than one year old and children undergoing emergency surgeries were excluded. Cases having active URTI, i.e., with fever, were cancelled for undergoing elective surgery, and thus excluded. The data was collected by history from parents, general and systemic examination, and by noting the type of surgery/anesthesia modality planned. The data was collected by the primary and co-investigator. General anesthesia was employed for all surgeries using intravenous fentanyl as an opioid, propofol as an induction agent, cisatracurium as a non-depolarizing muscle relaxant, sevoflurane and air or nitrous oxide for maintenance of general anesthesia, paracetamol as an analgesic, and neostigmine with glycopyrrolate for reversal of neuromuscular blockade. The choice of airway (endotracheal tube or laryngeal mask airway) was decided based on the type of surgery. A sample size calculation was not done because of the exploratory nature of the study and the absence of a gold standard rating scale for comparing results to. Therefore, the sample size was based on convenience. We planned to include and analyze the data of all children who fulfilled the inclusion criteria for six months from the day of starting.

Statistical analysis

Descriptive statistics were used to summarize patient demographics, surgical characteristics, and outcome variables. Continuous variables were reported as medians with interquartile ranges (IQR), and categorical variables were presented as counts with percentages. Between-group comparisons were performed using the Wilcoxon rank-sum test for continuous variables and Pearson’s chi-squared test or Fisher’s exact test for categorical variables, as appropriate.

To evaluate the predictive performance of TOTAL_SCORE for the binary outcome EVENTS_STATUS, we constructed a univariable logistic regression model. The predicted probabilities from the model were used to classify cases using a threshold of 0.5. Model performance was assessed using sensitivity, specificity, accuracy, and area under the receiver operating characteristic (ROC) curve (AUC). AUC and its 95% confidence interval (CI) were estimated using the DeLong method. ROC curves were visualized using the ‘ggroc’ function from the ‘pROC’ package. Confidence intervals for sensitivity, specificity, and accuracy were estimated via nonparametric bootstrapping (1,000 replicates). McNemar’s test was used to assess discordance in classification outcomes. All analyses were conducted using R (version 4.2.2), with statistical significance set at p < 0.05.

## Results

A total of 270 patients were included in the analysis, among whom 25 (9.3%) experienced postoperative events. These included desaturation (68%), laryngospasm (24%), and bleeding requiring re-exploration under general anesthesia (8%). The remaining 245 patients (90.7%) had no recorded postoperative events. The median TOTAL_SCORE was significantly higher in patients who experienced events (12.0; IQR: 12.0-14.0) compared to those who did not (10.0; IQR: 10.0-10.0), with a p-value < 0.001 (Table 1).

**Table 1:** Summary of demographic data and the analysis of the scores generated GA: general anesthesia, SD: standard deviation, TTR: tongue-tie release a: Unpaired t-test b: Pearson's Chi-squared test c: Wilcoxon rank sum test

Characteristic	Overall, N = 270	No Event, N = 245	Event, N = 25	p-value
Age	6.1 (±3.2)	6.2 (±3.2)	5.3 (±3.6)	0.11^a^
Gender				0.14^b^
F	113/270 (42%)	106/245 (43%)	7/25 (28%)	
M	157/270 (58%)	139/245 (57%)	18/25 (72%)	
Surgery				0.026^c^
Adenoidectomy	100/270 (37%)	93/245 (38%)	7/25 (28%)	
Adenoidectomy + myringotomy	2/270 (0.7%)	2/245 (0.8%)	0/25 (0%)	
Adenoidectomy with Myringotomy + Grommet insertion	3/270 (1.1%)	0/245 (0%)	3/25 (12%)	
Adenotonsillectomy	76/270 (28%)	67/245 (27%)	9/25 (36%)	
Myringotomy + Grommet insertion	7/270 (2.6%)	7/245 (2.9%)	0/25 (0%)	
Myringotomy and grommet insertion	18/270 (6.7%)	18/245 (7.3%)	0/25 (0%)	
Nasal endoscopy	1/270 (0.4%)	1/245 (0.4%)	0/25 (0%)	
Tongue Tie Release	1/270 (0.4%)	1/245 (0.4%)	0/25 (0%)	
Tonsillectomy	59/270 (22%)	53/245 (22%)	6/25 (24%)	
Tonsillectomy Myringotomy	1/270 (0.4%)	1/245 (0.4%)	0/25 (0%)	
TTR	2/270 (0.7%)	2/245 (0.8%)	0/25 (0%)	
C				<0.001^c^
1	241/270 (89%)	232/245 (95%)	9/25 (36%)	
2	28/270 (10%)	13/245 (5.3%)	15/25 (60%)	
5	1/270 (0.4%)	0/245 (0%)	1/25 (4.0%)	
O				<0.001^c^
1	250/270 (93%)	241/245 (98%)	9/25 (36%)	
2	17/270 (6.3%)	3/245 (1.2%)	14/25 (56%)	
5	3/270 (1.1%)	1/245 (0.4%)	2/25 (8.0%)	
L				<0.001^c^
1	257/270 (95%)	239/245 (98%)	18/25 (72%)	
2	3/270 (1.1%)	2/245 (0.8%)	1/25 (4.0%)	
5	10/270 (3.7%)	4/245 (1.6%)	6/25 (24%)	
D				>0.9^c^
1	1/270 (0.4%)	1/245 (0.4%)	0/25 (0%)	
2	2/270 (0.7%)	2/245 (0.8%)	0/25 (0%)	
5	267/270 (99%)	242/245 (99%)	25/25 (100%)	
S				0.088^c^
1	28/270 (10%)	28/245 (11%)	0/25 (0%)	
2	242/270 (90%)	217/245 (89%)	25/25 (100%)	
TOTAL_SCORE	10.24 (±1.23)	10.00 (±0.90)	12.64 (±1.50)	<0.001^c^
EVENTS				>0.9^c^
Bleeding-re-exploration done, GA again	2/25 (8.0%)	0/0 (NA%)	2/25 (8.0%)	
Desaturation	17/25 (68%)	0/0 (NA%)	17/25 (68%)	
Laryngospasm	6/25 (24%)	0/0 (NA%)	6/25 (24%)	
No event	245	245	0	

**Figure 1:**
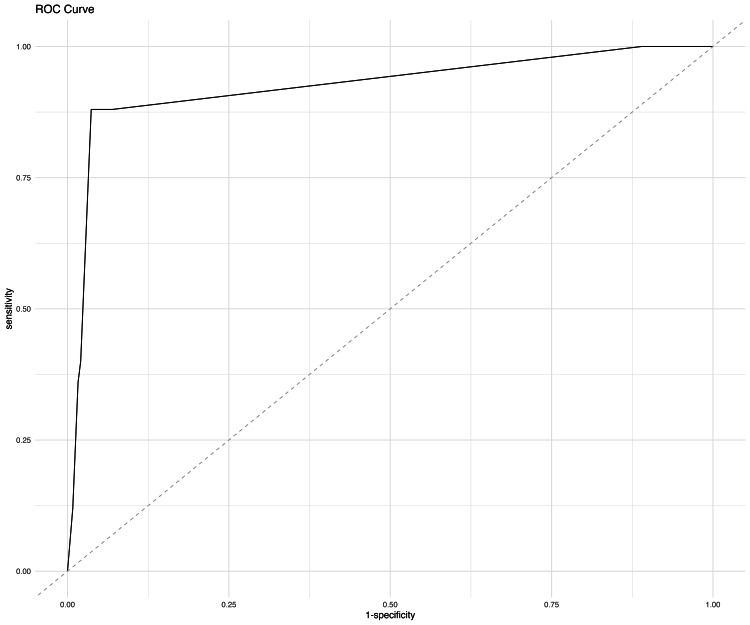
Receiver operating characteristic (ROC) curve

A logistic regression model using TOTAL_SCORE as the sole predictor of postoperative events (EVENTS_STATUS) demonstrated strong discriminatory ability, with an area under the receiver operating characteristic (ROC) curve (AUC) of 0.92 (95% CI: 0.86-0.99) (Figure 1). This indicates that the model was able to effectively distinguish between patients who did and did not experience postoperative complications based solely on their TOTAL_SCORE.

At a decision threshold of 0.5, the model correctly classified 92.6% of patients overall (accuracy: 92.6%, 95% CI: 89.3-96.0). The specificity of the model was high at 97.96% (95% CI: 95.9-99.6), indicating a strong ability to correctly identify patients without events. However, the sensitivity was lower at 40.0% (95% CI: 20.7-60.0), meaning the model detected only 40% of patients who experienced complications.

The positive predictive value (PPV) was 66.7%, suggesting that two-thirds of patients predicted to have an event did so. The negative predictive value (NPV) was 94.1%, indicating that patients predicted not to have events were indeed unlikely to experience complications. These values reflect the underlying low event prevalence (9.3%) and the model's conservative tendency to minimize false positives.

## Discussion

The COLDS score is a pre-anesthetic risk assessment tool developed by Lee and August to predict the likelihood of PRAEs in children with upper respiratory tract infections (URIs) [9]. Each component is assigned a value of 1, 2, or 5 based on the severity of the risk factor, and the total score ranges from 5 to 25, with a higher score suggestive of a higher propensity of PRAEs. The COLDS score was validated by Kim et al. in 2022 using a retrospective cohort of 6252 children who underwent elective surgery under general anesthesia [10]. The authors concluded that the COLDS score had a moderate predictive power for PRAEs, based on the receiver-operating characteristic curve (AUC) of 0.652. They also found that age, current symptoms, and the COLDS score were significant variables in predicting PRAEs. They suggested that the COLDS score could be used as a risk assessment tool for children with URIs undergoing elective surgery. Another study by Shaw et al. in 2018 aimed to optimize the COLDS score by changing the scoring key and weighting the components based on logistic regression [11]. They collected data on 536 children over six months and found that re-keying and reweighting the COLDS score improved the AUC to 0.70 and 0.71, respectively. They concluded that the COLDS score could be further improved by modifying the scoring system.

In an observational study involving 216 pediatric patients between the ages of 1-5 years undergoing ilioinguinal surgery, Jarraya et al. aimed to evaluate the validity of the COLDS score in predicting PRAEs [12]. In this study, the incidence of PRAE was 21%, and the various predictors of PRAE were underlying respiratory diseases, children who were passive smokers, cases that were postponed before 15 days, and patients having a COLDS score of more than 10. Egbuta and Mason reviewed all the risk factors and the various scores to predict PRAEs in pediatric surgical patients [13]. Along with other scores, they also reviewed the COLDS score. The authors mentioned that the COLDS score was less effective at anticipating the occurrence of laryngospasm and more useful in predicting bronchospasm, desaturation, the requirement for beta-agonist medication, and protracted cough. Also, the COLDS score could predict whether a planned surgical procedure will be cancelled due to anesthesia-related issues.

Shenoy et al. conducted a single-center, prospective, observational study comprising 270 children under two years of age undergoing elective cleft surgery to identify various predictors of PRAE and also to identify the most frequent PRAE [14]. The results revealed that the overall incidence of PRAE was 1.85%, with laryngospasm being the most common event. The authors concluded that children with higher COLDS scores and those who pose difficult intubation (based on Cormack-Lehane grade view ≥ 3) are more likely to develop PRAE following cleft repair surgery. Wudineh et al. investigated the prevalence and factors responsible for PRAE in 210 pediatric surgical patients [15]. The prevalence was 26.2%, with a total of 129 episodes of PRAEs, with desaturation being the most common one (47.3%). The factors responsible for PRAEs, based on the analysis, were age less than one year, recent history of active URTI, ASA-PS 3 or more, and airway surgeries. Stepanovic et al. comprehensively reviewed the existing evidence available for perioperative preparation in children with upper URTI, including COVID-19 infection [16]. The authors concluded that several factors could facilitate a smooth conduct of various pediatric surgeries. The factors mentioned were having an experienced anesthesia team managing the airway and involved in perioperative care, preoperative inhaled salbutamol if indicated, premedication with alpha 2 agonists, propofol induction, using a supraglottic airway device instead of a tracheal tube for airway management if feasible, considering total intravenous anesthesia for maintenance, and avoiding desflurane. The authors also commented that the involved anesthesiologists must ultimately decide on the best time for elective surgery and medical optimization based on case-by-case evaluations of each patient's risk of perioperative respiratory adverse events, even though risk stratification tools can help with decision-making.

Limitations

Despite the high specificity and overall accuracy, the moderate sensitivity indicates that some patients at risk for postoperative events may not be identified using TOTAL_SCORE alone. McNemar’s test for paired proportions showed a statistically significant discordance between predicted and actual classifications (p = 0.044), reflecting the model’s imbalanced performance - specifically, its higher rate of false negatives compared to false positives. Overall, while the model performs well in ruling out patients unlikely to have complications (high NPV and specificity), its ability to positively identify high-risk cases is more limited. These findings suggest that while TOTAL_SCORE is a strong general discriminator, additional clinical features may be needed to improve sensitivity and support preoperative risk stratification. We analyzed only pediatric patients undergoing ENT surgery only, which is another limitation. However, the reason for this was that these patients had chronic respiratory ailments and the surgery also involved the airway predominantly.

## Conclusions

Based on the analysis of the COLDS score data, we conclude that the COLDS score can comprehensively distinguish between patients who will and will not have postoperative respiratory complications based on the total score generated. However, in some patients, the score may not be able to identify patients at risk of respiratory adverse events, based on its moderate sensitivity. We suggest making decisions based on clinical assessment and also incorporating other scores to identify high-risk patients.
